# The Application of Buccal Fat Pad to Cover Lateral Palatal Defect Causes Early Mucolization

**DOI:** 10.7759/cureus.17532

**Published:** 2021-08-29

**Authors:** Iqra Khan, Namiya Cho, Mehtab Ahmed, Owais Ahmed, Mirza Shehab A Beg

**Affiliations:** 1 Plastic and Reconstructive Surgery, Liaquat National Hospital and Medical College, Karachi, PAK

**Keywords:** buccal fat pad, lateral palatal defect, mucolisation, cleft palate repair, cleft palate

## Abstract

Introduction

Cleft lip and cleft palate are among the most common birth defects. These deformities lead to profound psychosocial and functional effects on cleft palate patients. Several surgical techniques have been described for the repair of the cleft. The defects lateral to mucoperiosteal flaps closure are sometimes covered with sterile gauze soaked with soft paraffin or tincture of benzoin or are left open for mucolization by means of secondary intention. The buccal fat pad (BFP) is used as a pedicled graft to cover the exposed bone of the lateral palatal defect, and it is associated with proposed benefits of early healing and fewer effects on transverse growth of the maxilla.

Materials and methods

This was a prospective study involving 42 cleft palate patients who underwent cleft palate repair; 21 patients received BFP as an additional step to cover lateral palatal defect while the rest of the patients (n=21) underwent conventional surgical cleft palate repair and the defect was covered with Surgicel (Ethicon, Inc., Bridgewater, NJ). Postoperative follow-up was conducted at first, second, and third weeks postoperatively to assess the time required for mucolization.

Results

Our cohort of 42 patients included an equal number of complete and incomplete cleft palate patients. Follow-up at the first postoperative week showed an equal number (n=21, 100%) patients with incomplete mucolization on both groups, while at the second postoperative follow-up, only one (4.8%) of the patients who underwent conventional cleft palate repair had complete mucolization while 20 (95.2%) among the patients who underwent BFP had complete mucolization. At the third-week postoperative follow-up, three (14.3%) patients from the conventional group had complete mucolization, while 18 (85.7%) had incomplete mucolization. Only two patients (4.8%) developed recipient area complications, and they were managed conservatively.

Conclusion

BFP is a good source of vascularized tissue to cover the hard palate bones after primary cleft repair. It is easy to harvest as a local tissue with a low learning curve. The epithelialization rate is faster than conventional methods with minimal complication rates.

## Introduction

Cleft lip and cleft palate are among the most common birth defects with an incidence of 1.91 per 1000 births. According to extrapolated statistics revealed by Smile Train, Pakistan ranks fourth in the number of clefts births in the world after China, India, and Indonesia [1]. These defects result in profound psychosocial and functional effects on cleft palate patients. This calls for an imminent need for intervention in such patients at the age of 6-12 months [2] to help them develop normal speech at appropriate age [3].

Several surgical techniques have been described for the repair of the cleft palate, including von Langenbeck, Bardach’s, Veau/Wardill/Kilner, and Furlow’s techniques [4,5]. The goal of the surgery is to restore normal anatomy, which helps in feeding to prevent nasal regurgitation and prevention of articulation issues secondary to the removal of air through the nose [6]. The techniques used to close the defect may result in bare bony areas on the hard palate after the elevation of mucoperiosteal flaps and their subsequent medial mobilization [7].

The defect lateral to mucoperiosteal flaps closure is sometimes covered with sterile gauze soaked with soft paraffin or tincture of benzoin or are left open for mucolization by means of secondary intention [8]. Re-epithelialization or mucolization of these defects takes three to four weeks, and the defect usually heals by secondary intention and scarring that can affect the transverse growth of the maxilla [9].

The buccal fat pad (BFP) is used as a pedicled graft to cover the exposed bone of the lateral palatal defect, and it is associated with proposed benefits of early healing and fewer effects on the transverse growth of the maxilla [9]. The BFP is an encapsulated fatty tissue composed of a central body and four projections: buccal, pterygoid, pterygopalatine, and temporal [3], as described by Egyedi [10]. It is distinct from subcutaneous fat in the buccal space [11]. The blood supply of the BFP is derived from the facial and maxillary arteries. Studies have reported a high success rate of healing in procedures that use BFP as a vascularized graft due to its rich vascularity, closeness to the treatment area, low donor-site morbidity, and technically simple surgical procedure for covering nearby defects [12]. Multiple studies have shown good results with the use of a pedicled BFP showing early epithelialization within three to four weeks after surgery by providing an additional vascular layer to promote healing [13-14].

The aim of this study was to evaluate the effects of healing on bare bony palatal bones after the use of BFP and to compare the results of epithelialization between a group that underwent the BFP procedure and one that was treated with conventional surgical cleft palate repair.

## Materials and methods

This was a prospective study involving 42 patients who underwent cleft palate repair. It was conducted in the Department of Plastic and Reconstructive Surgery at the Liaquat National Hospital and Medical College after taking approval from the Ethical Review Board of the Liaquat National Hospital. All patients were examined by the consultants of the department. Patients were selected after a thorough history and clinical examination. The group comprised 24 male and 18 female children; a total of 42 patients aged between 8-14 months were included. The operation was done under general anesthesia, after obtaining full informed consent. Intravenous metronidazole and amoxicillin/clavulanic acid were administered prophylactically before the surgery. Out of the total 42 patients, 21 received BFP as an additional step while the remaining 21 patients underwent conventional surgical cleft palate repair. The inclusion criteria were as follows: patients with clinically diagnosed cleft palate, either unilateral or bilateral, with both complete and incomplete cleft palates.

Postoperative follow-up was conducted at the first, second, and third weeks postoperatively, which included clinical evaluation and examination of patients. It was advised that patients should be given a clear liquid diet and was encouraged to drink water after every meal. Data analysis was performed using SPSS Statistics for Windows, version 23 (IBM, Armonk, NY). Stratification was done based on postoperative follow-up complications rates by using the Chi-square test, and a p-value less than 0.05 was considered statistically significant.

Surgical technique

**Figure 1:**
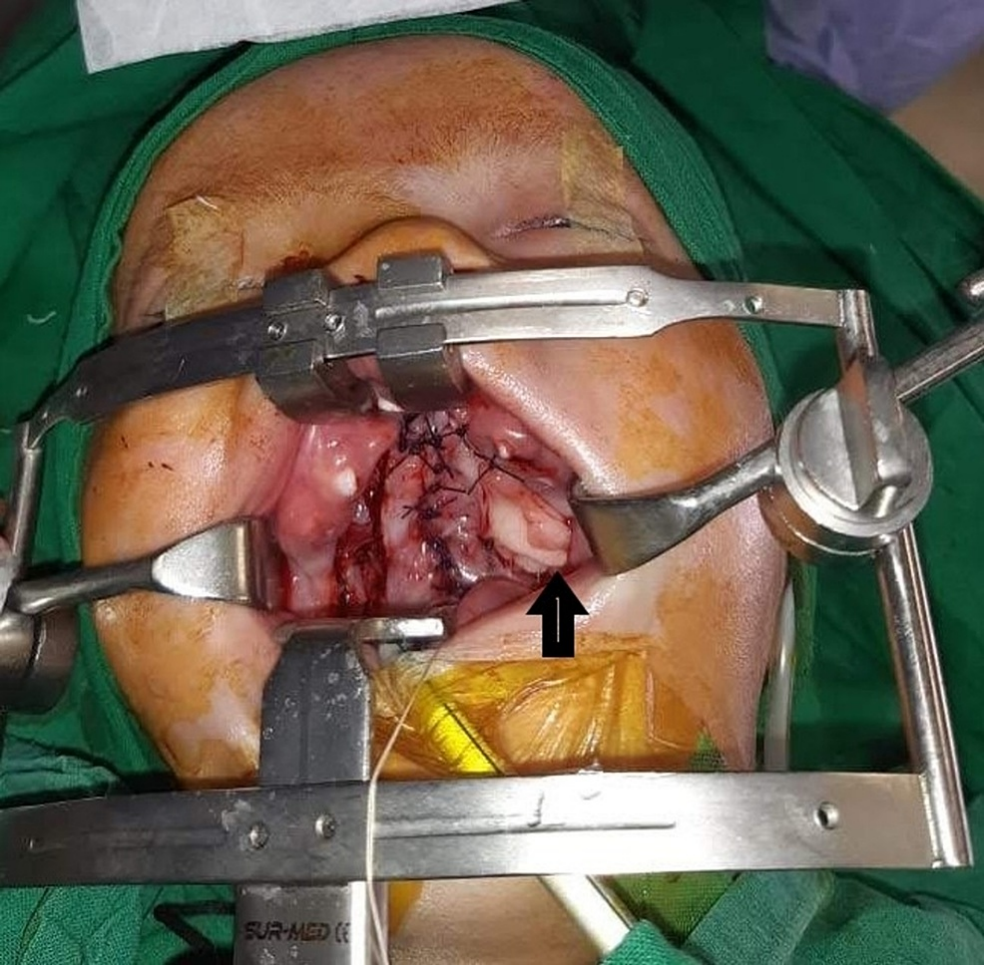
Cleft palate repair with buccal fat pad for lateral palatal defects

The surgical technique for BFP essentially follows the dissection planes for standard palate repair techniques. Lateral releasing incisions are given along with the dissection of oral and nasal layers in the midline. Levator muscle is dissected from abnormal attachment along the posterior border of the hard palate. Nasal layer repair is done followed by the approximation of muscle in the midline. Oral layer repair is then completed. At the end of the procedure, the extent of lateral defects is inspected to get an idea of the amount of fat pad required to fill the defect. The lateral releasing incision is extended posteriorly towards the bulge of the fat pad on the oral mucosa. Gentle dissection with scissors is done until the fat starts to appear in the wound; this fat is gently and gradually pulled out with forceps, avoiding excessive stretch and tension to prevent damage. When enough fat pad is mobilized, it is used to fill the lateral defect and stabilized with vicryl sutures. Care should be taken to avoid excessive stretch on fat when stabilizing, and it should be ensured that it does not hang in the air so that it fills and occupies the space of lateral defect in its floor. The opposite lateral defect is filled with an absorbable hemostat (Surgicel; Ethicon, Inc., Bridgewater, NJ) (Figure 1).

## Results

Out of the total 42 patients, an equal number of complete cleft palate patients (18, 85.7%) were included in both the conventional palate repair and BFP groups, while only three (14.3%) patients in each group had incomplete cleft palates. During the follow-up, 95.2% of patients developed no complications related to either donor or recipient site; only two patients (4.8%) developed recipient area complications. Initial follow-up data of the first postoperative week showed an equal number (21, 100%) patients with incomplete mucolization in both groups, while at the second postoperative follow-up, only one (4.8%) of the patients who underwent conventional cleft palate repair had complete mucolization while 20 (95.2%) among the patients who underwent BFP had complete mucolization. At the third postoperative week follow-up, three (14.3%) patients from the conventional group had complete mucolization, while 18 (85.7%) had incomplete mucolization. Similar outcomes were noted as 20 (95.2%) patients in both groups developed no complications while one (4.8%) patient in each group developed recipient site complications including wound dehiscence; they were managed conservatively.

The Chi-square and Fisher's exact tests were used to find out the association between categorical variables. We found a statistically significant association of the study group with the mucolization status on day 14 (p<0.001) and mucolization status on day 21 (p<0.001), while statistically insignificant associations of the study group were found with respect to gender (p=1.000), age group (p=1.000), side of cleft (p=1.000), type of cleft (p=1.000), and complications (p=1.000).

The side of cleft was insignificantly associated with the mucolization status on day 14 (p=1.000), mucolization status on day 21 (p=1.000), and complications (p=0.489). The type of cleft was also insignificantly associated with the mucolization status on day 14 (p=1.000), mucolization status on day 21 (p=0.685), and complications (p=1.000). Detailed results of the mucolization status are presented in Table 1.

**Table 1:** Mucolization status on different postoperative weeks

Type of cleft	Postoperative week 1, n (%)	Postoperative week 2, n (%)	Postoperative week 3, n (%)
Complete/incomplete palate	36 (85.7%)/6 (14.3%)	36 (85.7%)/6 (14.3%)	36 (85.7%)/6 (14.3%)
Unilateral/bilateral	22 (52.4%)/21 (47.6%)	22 (52.4%)/21 (47.6%)	22 (52.4%)/21 (47.6%)
Incomplete/complete mucolization	42 (100%)/0 (0%)	21 (50%)/21 (50%)	18 (42.9%)/24 (57.1%)

## Discussion

Cleft palate is one of the most commonly encountered congenital anomalies in the head and neck region [6]. Various procedures are used to close this oronasal communication and to restore the normal anatomy of the palate. The use of a buccal fat flap as an adjunct to cover the denuded bones is in an evolving stage. It provides an additional vascular layer that promotes healing and decreases the time interval from surgery to epithelialization of hard palate bones after lateral relaxing incisions. The advantage of using this flap intraorally is that it does not require a skin graft, as it will epithelialize.

The BFP develops early in utero and is a mature tissue at the time of cleft palate repair. It is an encapsulated fatty tissue, syssarcosis type [3], with an average thickness of 6 mm, and provides adequate tissue to cover lateral palatal defects [15]. In our study, the intraoral horizontal incision was used to harvest the flap and it may prevent the hindrance on the maxillary growth; however, this was not confirmed in our study either by radiography or any other kind of assessment.

As per Ayyash et al., it has been thought that this flap prevents abnormal movement of the levator muscle sling, thereby aiding the cleft palate repair to be tension-free and reducing the rate of palatal fistula formation [15]. Levi et al. have described the use of buccal fat flaps to cover areas of denuded bone in cleft palate repair with the goal of preventing healing by secondary intention and subsequent maxillary growth restriction [7].

The rate of epithelialization of the graft and complete coverage of the lateral palatal defects were the criteria for the successful outcome of the study. As per multiple studies, the healing of pedicled graft takes four to six weeks [14,16], which is in contrast with our study, which showed epithelialization of the defect in three weeks. This process of early healing may be due to the rich blood supply and newly formed wound surrounding the defect.

The beneficial aspects for considering BFP include easy availability, good blood supply, ease to harvest, the low learning curve of the surgical technique, and the absence of donor-site morbidity. There is no relationship between its size or volume and age and body weight. Additional data are required to draw definitive conclusions about the efficacy and mechanism of buccal fat flaps. As per Ruslin et al., the failure of the procedure and the complication rates seen were very low [14], which align with our findings. 

Various studies have shown the procedure having minimal effect on maxillary growth and facial aesthetic impact on the donor site [17]. The disadvantages of its use are that it can be used as a source of vascularised local tissue for secondary reconstruction of cleft palate [18,19], and the restriction in mouth opening [15].

The limitations of the study include the fact that it was a single-center study with a limited number of patients. There is a need to conduct multicenter randomized controlled trials with longer durations of follow-up.

## Conclusions

BFP is a good source of vascularized tissue to cover the hard palate bones after primary cleft repair. It is easy to harvest as it is a local tissue with a low learning curve. The epithelialization rate is faster compared to conventional methods, with minimal complication rates.
